# Expression Profiling of Human Immune Cell Subsets Identifies miRNA-mRNA Regulatory Relationships Correlated with Cell Type Specific Expression

**DOI:** 10.1371/journal.pone.0029979

**Published:** 2012-01-20

**Authors:** Florence Allantaz, Donavan T. Cheng, Tobias Bergauer, Palanikumar Ravindran, Michel F. Rossier, Martin Ebeling, Laura Badi, Bernhard Reis, Hans Bitter, Matilde D'Asaro, Alberto Chiappe, Sriram Sridhar, Gonzalo Duran Pacheco, Michael E. Burczynski, Denis Hochstrasser, Jacky Vonderscher, Thomas Matthes

**Affiliations:** 1 Pharma Research and Early Development, Translational Research Sciences, F. Hoffmann-La Roche Ltd., Basel, Switzerland; 2 Pharma Research and Early Development, Translational Research Sciences, Roche, Nutley, New Jersey, United States of America; 3 Department of Genetics & Laboratory Medicine, University Hospital of Geneva, Geneva, Switzerland; 4 Hematology Service, University Hospital of Geneva, Geneva, Switzerland; Kyushu Institute of Technology, Japan

## Abstract

Blood consists of different cell populations with distinct functions and correspondingly, distinct gene expression profiles. In this study, global miRNA expression profiling was performed across a panel of nine human immune cell subsets (neutrophils, eosinophils, monocytes, B cells, NK cells, CD4 T cells, CD8 T cells, mDCs and pDCs) to identify cell-type specific miRNAs. mRNA expression profiling was performed on the same samples to determine if miRNAs specific to certain cell types down-regulated expression levels of their target genes. Six cell-type specific miRNAs (miR-143; neutrophil specific, miR-125; T cells and neutrophil specific, miR-500; monocyte and pDC specific, miR-150; lymphoid cell specific, miR-652 and miR-223; both myeloid cell specific) were negatively correlated with expression of their predicted target genes. These results were further validated using an independent cohort where similar immune cell subsets were isolated and profiled for both miRNA and mRNA expression. miRNAs which negatively correlated with target gene expression in both cohorts were identified as candidates for miRNA/mRNA regulatory pairs and were used to construct a cell-type specific regulatory network. miRNA/mRNA pairs formed two distinct clusters in the network corresponding to myeloid (nine miRNAs) and lymphoid lineages (two miRNAs). Several myeloid specific miRNAs targeted common genes including ABL2, EIF4A2, EPC1 and INO80D; these common targets were enriched for genes involved in the regulation of gene expression (*p*<9.0E-7). Those miRNA might therefore have significant further effect on gene expression by repressing the expression of genes involved in transcriptional regulation. The miRNA and mRNA expression profiles reported in this study form a comprehensive transcriptome database of various human blood cells and serve as a valuable resource for elucidating the role of miRNA mediated regulation in the establishment of immune cell identity.

## Introduction

Blood is a complex tissue consisting of several cell types with unique functions and correspondingly, distinct gene expression profiles. Using genome wide gene expression profiling by microarray, several groups have succeeded in characterizing the gene expression profiles of selected immune blood cell populations, including T cells [1], dendritic cells [2], monocytes [3], B cells [4], megakaryocytes, erythroblasts and platelets [5]. Several groups have also isolated and profiled cell subsets from healthy donors' blood to identify immune cell-specific mRNA transcripts [6], [7], [8]. Determining the specificity of mRNA expression profiles in blood cells is clearly important for both understanding the biology of the immune and hematopoietic systems [9] and for characterizing blood as an important source of transcriptional biomarkers [10]. However, these profiles represent only a snapshot of gene expression in blood cells – of greater interest are the dynamic changes in transcriptional regulation that occur during the differentiation process, which ultimately leads to gene expression patterns observed in the differentiated cells. Transcription factors are classic examples of such transcriptional regulators; the role of transcription factors in haematopoiesis for example has been demonstrated in several well-characterized systems, including T-bet, STAT6, FOXp3 and RORgt in the regulation of T helper cell differentiation.

Besides transcription factors, there is increasing recognition that non-protein-coding RNAs can play an equally important role as modulators of gene expression, which may also impact the immune cell differentiation process. These regulatory RNAs include small nucleolar RNAs (snoRNAs), small Cajal body specific RNAs (ScaRNA) and micro-RNAs (miRNA). snoRNAs are a class of small RNA molecules that primarily guide chemical modifications of other RNAs, mainly ribosomal RNAs, transfer RNAs and small nuclear RNAs. There are two main classes of snoRNA, the C/D box snoRNAs, which are associated with methylation, and the H/ACA box snoRNAs which are associated with pseudouridylation. scaRNAs are a specific class of small nucleolar RNAs that localize to the Cajal bodies and guide the modification of RNA polymerase II transcribed spliceosomal RNAs U1, U2, U4, U5 and U12 [11]. miRNAs are small (∼21mer) regulatory RNA molecules encoded in plant and animal genomes that regulate the expression of target genes by binding to the 3′ untranslated regions of specific mRNAs, thereby triggering mRNA degradation or translational repression. Of the regulatory RNA classes, miRNAs have been shown to play an important role in hematopoiesis and in the immune response ([12], [13], reviewed in [14]), but their relative expression across different cells of the immune system is not well described. Although previous studies have measured expression of miRNAs in selected immune cell systems, i.e. B cells [15], NK cells [16], DCs [17], T lymphocyte development [18] and differentiation of naive T cells into effector and memory T cell subsets [19], systematic efforts to profile global miRNA expression across a diverse panel of immune cell subsets are lacking.

To characterize more precisely the role of miRNAs in different immune cells, we performed genome wide miRNA expression profiling for nine immune cell subsets (neutrophils, eosinophils, monocytes, B cells, NK cells, CD4 T cells, CD8 T cells, myeloid dendritic cells (mDCs) and plasmacytoid dendritic cells (pDCs)), isolated from whole blood collected from multiple human donors. We complemented these data by performing genome wide mRNA expression profiling on samples from the same donors to enable accurate identification of genes targeted by specific miRNAs. Based on growing evidence that many miRNAs cause mRNA degradation or instability [20], simultaneously measured expression profiles of miRNAs and the mRNAs they target for degradation should show an inverse relationship, i.e. a negative correlation. We identified mRNAs that were both negatively correlated with miRNA expression and also computationally predicted to be miRNA targets. In addition, we validated and enhanced the robustness of the miRNA signatures discovered by performing a similar experiment on an independent cohort recruited at the University Hospital of Geneva (HUG). This yielded a highly confident set of mRNA and miRNA transcripts specific to each of the studied cell populations. Finally, we used both the mRNA and miRNA expression profiles to construct a regulatory network for miRNA-mediated gene expression in immune blood cells. Our study is unique in its systematic approach towards the characterization of miRNA and mRNA expression across various immune cell subsets, and identifies potential miRNA-mRNA regulatory relationships that may be relevant for immune cell functions and establishment of their respective transcriptomic identities.

## Results

### Identification of cell-type specific miRNA transcripts

Nine cell subsets (CD16+CD66b+ Neutrophils, CD16−CD66b+ Eosinophils, CD14+ Monocytes, CD4+ T cells, CD8+ T cells, CD56+ NK cells, CD19+ B cells, CD123+ pDCs and CD11c+ mDCs) were isolated from healthy human blood obtained from five pools of five donors each with the exception of monocytes, where 10 pools were used (Table S1, the donor demographic information is available in Table S2). Subjects were randomized across pools for age and gender. Purity of each isolated cell subset was greater than 95%, as assessed by flow cytometry, except for monocytes, CD4 T cells and mDCs for which the average purity was 94%, 92% and 91% respectively. Since pooling blood samples could theoretically cause cell activation, samples were kept at 4°C and were pooled only immediately before cell subset separation in order to minimize this risk. In fact, we have chosen to pool samples in order to get sufficient cell numbers for rare cell types (i.e. dendritic cells representing less than 1% of the PBMCs) and to decrease sample variability due to donor-to-donor variation.

To identify cell-type specific small non-coding RNA transcripts, total RNA was extracted from pooled samples, labeled and hybridized to Affymetrix miRNA chips to measure miRNA, snoRNA and scaRNA expression. To ascertain that pooling of samples had a minimal effect on miRNA expression, we isolated in parallel selected immune cell subsets (CD4, CD8, B cells, NK cells, neutrophils, eosinophils, monocytes) from single donors and compared their miRNA profile with the profile from pooled samples. Clustering samples by similarity of miRNA expression, samples from the same cell subsets clustered together independently of their origin (pooled or single donors). (Figure S1) The total number of expressed miRNAs was also similar between pooled and single donor samples, suggesting that pooled samples did indeed not differ significantly in their miRNA expression from single donor samples.

Using data from the pooled samples, 170 of the 7815 human-specific probesets displayed an average log_2_ signal value greater than 7 in at least one cell type and were deemed to be expressed above background. Probesets were assessed for differential expression across cell types using ANOVA, and were classified as specific to one, two or three cell types, based on hierarchical pair-wise comparisons (see methods). Few transcripts were found uniquely specific for either CD4+ T cells or CD8+ T cells when considered as individual cell types, and thus subsequent analyses considered these two T cell types as a single group “T cells” (see Methods).

**Figure 1:**
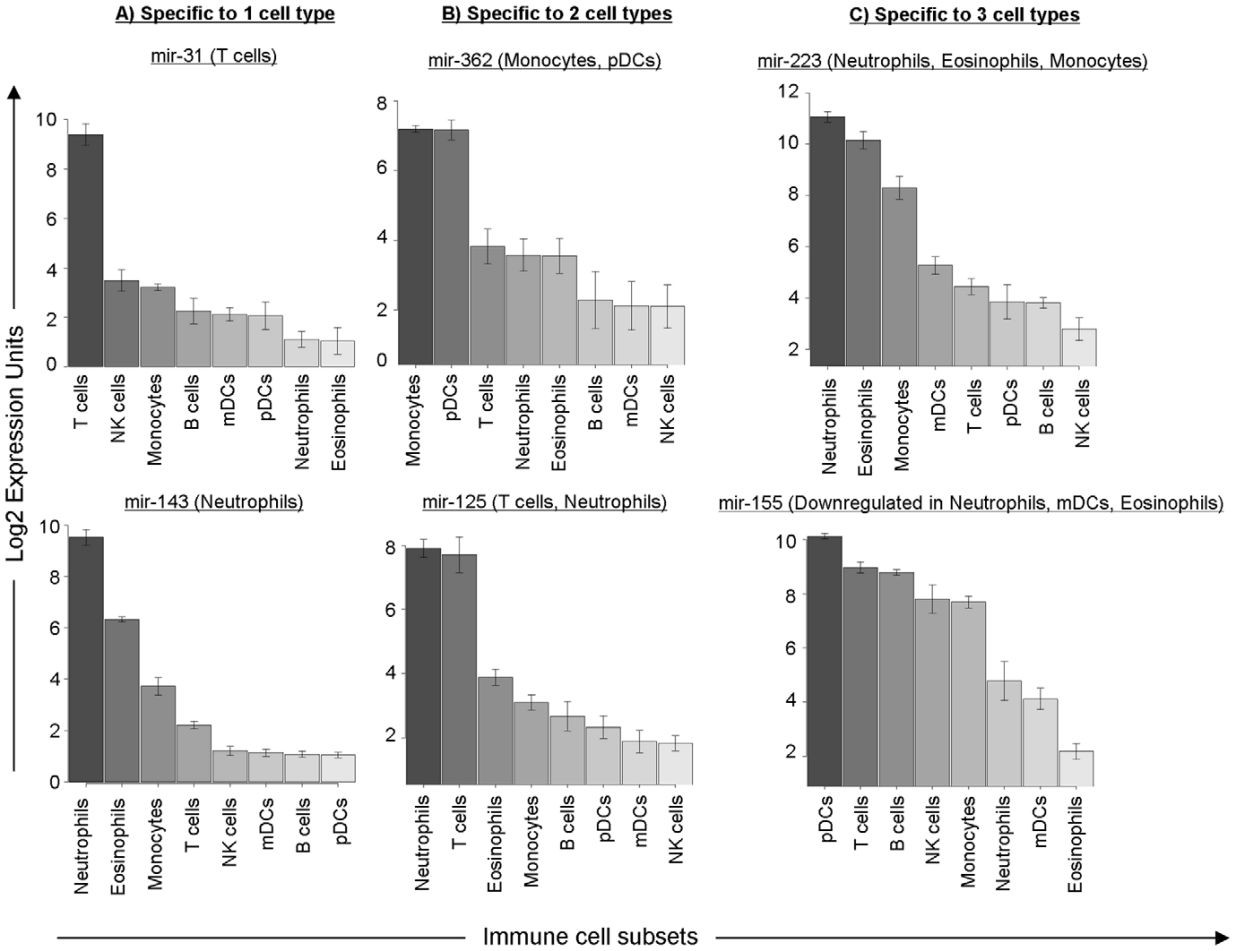
Cell type specific expression of miRNAs. miRNAs were grouped based on specificity to one, two or three cell types. **A**) miR-143 and miR-31 were specific to neutrophils and T cells respectively, while **B**) miR-362 and miR-125 were specific to monocytes, pDCs and T cells, neutrophils. **C**) miR-223 was specific to myeloid lineage cells (neutrophils, eosinophils and monocytes), whereas miR-155 was specific to lymphoid lineage cells (pDCs, T cells, B cells and NK cells).

Four small-RNA transcripts were each found to be expressed specifically in one cell type: miR-378 in monocytes, miR-31 in T cells, miR-935 in eosinophils and miR-143 in neutrophils (Figure 1). Five small-RNA transcripts were specifically expressed in two cell types, whereas nine transcripts were specifically expressed in three cell types (Table 1). miR-31 and miR-143 were verified to be specifically expressed in T cells and neutrophils in the single donor data (Figure S2). For the miRNAs unique to one cell type, miR-378 was found to be 4 fold up-regulated in monocytes relative to T cells, the cell type in which miR-378 had the second highest expression level (p*<0.04). miR-31 was expressed uniquely in T cells (at similar levels in CD4 and CD8 T cells) and was 59.2 fold up-regulated in T cells vs NK cells, the next highest expressing cell type (p* = 9.9e-9). miR-935 was 5.6 fold up-regulated in eosinophils compared to mDCs (p* = 0.007). Finally, we observed that miR-143 was uniquely expressed in neutrophils, the only cell type where the log2 RMA expression value was greater than 7. miR-143 was also 9.2 fold more expressed in neutrophils than in eosinophils; p* = 0.0007.

**Table 1:** miRNAs are specifically expressed in 1, 2 or 3 blood cell subsets.

Affymetrix ID	Specific Cell Type	Fold Change	p*	Log2 Expression
hsa-miR-378_st	Monocytes	3.96	4.37E-02	9.4
hsa-miR-31_st	T cells	59.23	9.90E-09	9.39
hsa-miR-935_st	Eosinophils	5.64	7.44E-03	7.67
hsa-miR-143_st	Neutrophils	9.16	7.45E-04	9.53
hsa-miR-362-5p_st	Monocytes; pDCs	10.18	1.31E-03	7.20; 7.17
hsa-miR-532-5p_st	Monocytes; pDCs	5.78	1.09E-02	7.88; 8.85
hsa-miR-500-star_st	Monocytes; pDCs	4.68	1.77E-02	7.32; 8.58
hsa-miR-663_st	B cells; NK cells	5.71	1.37E-05	9.35; 9.91
hsa-miR-125a-5p_st	T cells; Neutrophils	14.09	4.85E-06	7.7; 7.9
hsa-miR-150_st	B cells; T cells; NK cells	9.38	1.32E-03	11.74; 12.91; 11.61
HBII-239_st	B cells; T cells; NK cells	2.76	3.26E-03	9.06; 8.91; 8.68
HBII-429_st	B cells; T cells; NK cells	2.72	3.57E-02	9.85; 9.79; 9.00
HBII-202_st	B cells; T cells; NK cells	2.16	1.48E-03	11.3; 11.31; 11.02
U27_st	B cells; T cells; NK cells	2.16	4.33E-02	8.76; 8.46; 8.10
U95_st	B cells; T cells; NK cells	2.03	1.60E-04	12.16; 12.18; 12.01
hsa-miR-768-5p_st	B cells; T cells; NK cells	1.95	2.89E-02	12.47; 12.36; 12.52
hsa-miR-223_st	Monocytes; Eosinophils; Neutrophils	8.05	2.21E-02	8.29; 10.16; 11.06
hsa-miR-652_st	Monocytes; Eosinophils; Neutrophils	3.16	4.08E-02	8.46; 9.42; 8.59

The fold-change is between the highest expressing cell population relative to the next highest expressing cell population (p* is the ANOVA p-value for significance of this comparison, FDR corrected for multiple testing). The Log2 expression is the average expression observed in the different cell types in the order given in column 3 (specific cell type).

miR-362 and miR-125 were specific to two cells types. miR-362 was 10 fold more expressed in monocytes and pDCs than in the next highest expressing cell type, T cells. Similarly, miR-125 was expressed in neutrophils and T cells, 14.1 fold higher than in eosinophils. Small RNA transcripts specific to three cell types were expressed in cell types belonging to a common lineage: they were either lymphocyte specific (expressed in B cells, T cells and NK cells) or myeloid specific (expressed in monocytes and granulocytes). For the myeloid lineage, we observed miR-223 to be expressed only in monocytes and granulocytes (fold change = 8.1 between monocytes and mDCs; p* = 0.02). Like miR-223, miR-652 was also only expressed in myeloid lineage cells (monocytes and granulocytes, fold change = 3.2; p* = 0.04). For the lymphoid lineage, miR-150 was expressed mainly in B cells, T cells and NK cells (fold-change = 9.4 between NK cells and eosinophils, p* = 0.001). In samples from single donors, miR-150 and miR-223 were confirmed to be specifically expressed in lymphoid and myeloid lineage cells respectively (Figure S2). Interestingly, we observed that miR-155 was specifically expressed in lymphoid lineage cells but down-regulated in neutrophils, eosinophils and mDCs. This is consistent with previous reports that miR-155 is elevated in lymphocytic leukemias and enhances inflammatory T cell development [21], [22]. We observed other lymphocyte-specific small RNAs, including several snoRNAs like HBII-239 (predicted to guide the 2′O-ribose methylation of 5.8S rRNA on residue U14 [23]), as well as U27 and HBII-429, which have been shown to have miRNA-like activities [24]. However, their exact function in lymphoid cells remains to be determined.

### Identification of mRNA transcripts regulated by cell type specific miRNAs

We then set out to determine if any of the identified cell-type specific miRNAs, which regulate the expression of protein-coding genes, give rise to cell-type specific mRNA expression patterns. As a first step, we performed whole genome mRNA expression profiling on the same set of samples using Affymetrix HG-U133 Plus 2.0 microarrays. Data was normalized by RMA, and expression data from 3 samples (2 neutrophils, 1 eosinophil) were excluded because they showed a strong outlier expression profile as observed after a principal component analysis (data not shown). Probesets were additionally checked for sequence identity against current genomic annotation. Probesets that did not map to unique positions in the genome were discarded as an additional quality control measure. Similar to the miRNA data, mean probeset expression was compared across cell-types, and significance assessed using an ANOVA test. Probesets were judged to be specifically up or down-regulated in one, two or three cell types, if the expression level in the specific cell type(s) was significantly greater or lesser than expression in all the other cell types (criteria: p*<0.05, corrected for multiple testing by method of Benjamini-Hochberg and absolute fold change >4). Probesets up-regulated in one, two or three cell types were required to be expressed with an average log_2_ RMA signal value >7 in those respective cell types. Similarly, down-regulated probesets were required to be expressed with a signal value >7 in all other cell types.

**Figure 2:**
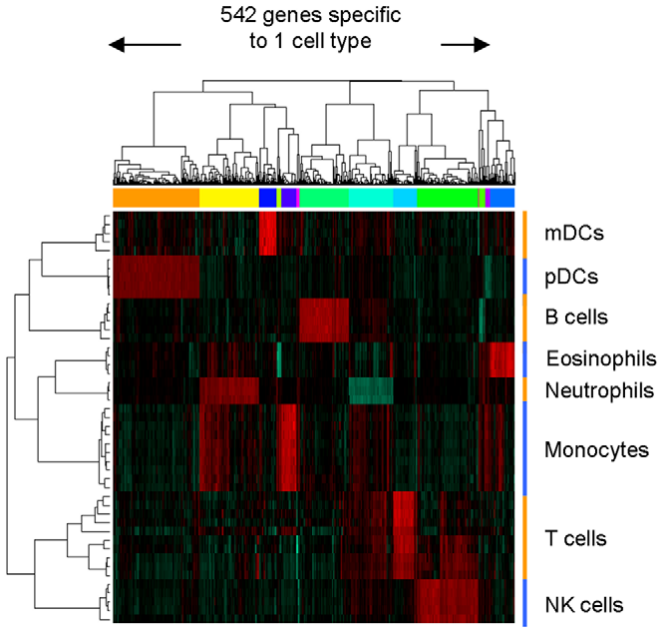
542 mRNA transcripts are uniquely up or down-regulated in one cell type. 696 genes were specifically up or down-regulated in one, two or three different cell-types, with the majority of genes (542) uniquely up or down-regulated in a single cell-type. Transformed expression levels are indicated by color scale, with red representing relative high expression and green relative low expression.

Using this approach, we identified 696 genes specifically up- or down-regulated in one, two or three different cell types (Table 2). Among those, 542 were specific to one cell type (456 up-regulated and 86 down-regulated), 109 were specific to two cell types (73 up-regulated and 36 down-regulated) and 45 were specific to 3 cell types (34 up-regulated and 11 down-regulated). Expression profiles for the 542 genes specific to single cell types were subject to hierarchical clustering (complete linkage using Pearson correlation as distance) and the results visualized in a heatmap in Figure 2. The full list is available in Table S3. Similar to the miRNA analysis, we compared again mRNA expression data from single donor samples to pooled samples to exclude pooling effects. Expression profiles of single and pooled donor samples were very similar with an average correlation >0.62 (Figure S3) and samples co-clustered in hierarchical clustering by cell-type rather than sample source (pooled or single donor) (Figure S4), suggesting that pooling effects on mRNA expression were indeed minimal.

**Table 2:** 696 transcripts are uniquely expressed by 1, 2 or 3 blood cell subsets.

	Number of up-regulated transcripts	Number of down-regulated transcripts	Total numbers of specific transcripts
1 cell type	456	86	542
2 cell types	73	36	109
3 cell types	34	11	45
Total	563	133	696

Interestingly, the distribution of genes specifically up-regulated in single cell types was evenly distributed across cell types ranging from 117 genes in pDCs to 21 genes in monocytes (Table 3), genes down-regulated in single cell types showed a different distribution: of the 86 down-regulated genes, a disproportionate majority of genes were repressed in neutrophils (61 genes; 71% of all downregulated genes) in contrast to 0% in monocytes. Genes down-regulated in two or three cell types followed a similar trend, where 30 of 47 genes identified were specifically repressed in neutrophils, suggesting that cell-type specific repression of mRNA transcripts occurs predominantly in end-differentiated, mature neutrophils.

**Table 3:** 542 uniquely expressed transcripts display distinct distributions across cell types, comparing up vs. down-regulated genes.

Cell Type	Number of up-regulated genes	Number of down-regulated genes
Neutrophils	84 (18.4%)	61 (70.9%)
Eosinophils	32 (7.0%)	5 (5.8%)
Monocytes	21 (4.6%)	0 (0%)
mDCs	24 (5.2%)	1 (01.1%)
pDCs	117 (25.7%)	10 (11.6%)
T lymphocytes	29 (6.4%)	2 (2.3%)
B lymphocytes	66 (14.5%)	5 (5.8%)
NK cells	83 (18.2%)	2 (2.3%)
Total	456	86

Next, we asked if any mRNA transcripts were significantly negatively correlated with miRNA expression and at the same time predicted to be miRNA targets by seed site matching algorithms. Pair-wise correlations were computed for 1,801 human miRNA probes and 20,367 curated mRNA probesets; the significance of correlation was assessed using a cutoff of −0.55 (False discovery rate (FDR) 1%, based on 100 permutations). In parallel, we used a modified version of the TargetScan algorithm (conservation assessed across human, dog, horse, cow, mouse, rat, opossum, excluding chicken) to determine miRNA targets by seed site matching. TargetScan predictions were further cross-checked against other computational (miRanda and miRDB) and experimental databases (mir2disease, tarbase). TargetScan predictions confirmed by at least one other database were used as the final list of predicted miRNA targets. We assessed the degree of overlap between mRNAs negatively correlated with a given miRNA and mRNAs predicted to be targets of individual miRNAs. We identified 94 miRNAs (corresponding to 79 miRNA families, annotation from mirDB) with significant overlap (FDR 5%, 100 permutations, see methods) (Table S4). Six of these miRNAs (miR-223, miR-143, miR-150, miR-500, miR-652 and miR-125) were cell-type specific based on our previous analysis. Interestingly, four of the six miRNAs were specific to cells of the myeloid lineage (monocytes, neutrophils and eosinophils), consistent with our earlier finding that most cell-type specific gene repression occurs in granulocytes (Table 4).

**Table 4:** 6 cell-type specific miRNAs are negatively correlated with significant numbers of target mRNAs as predicted by TargetScan.

Affymetrix ID	Num. Target mRNAs (TargetScan, negatively correlated)	Cell Type specificity
HSA-MIR-223	82	Monocytes; Eosinophils; Neutrophils
HSA-MIR-143	60	Neutrophils
HSA-MIR-150	27	B cells; T cells; NK cells
HSA-MIR-500	25	Monocytes; pDCs
HSA-MIR-652	11	Monocytes; Eosinophils; Neutrophils
HSA-MIR-125A	9	T cells; Neutrophils

### Validation of predicted miRNA target genes using external data

**Figure 3:**
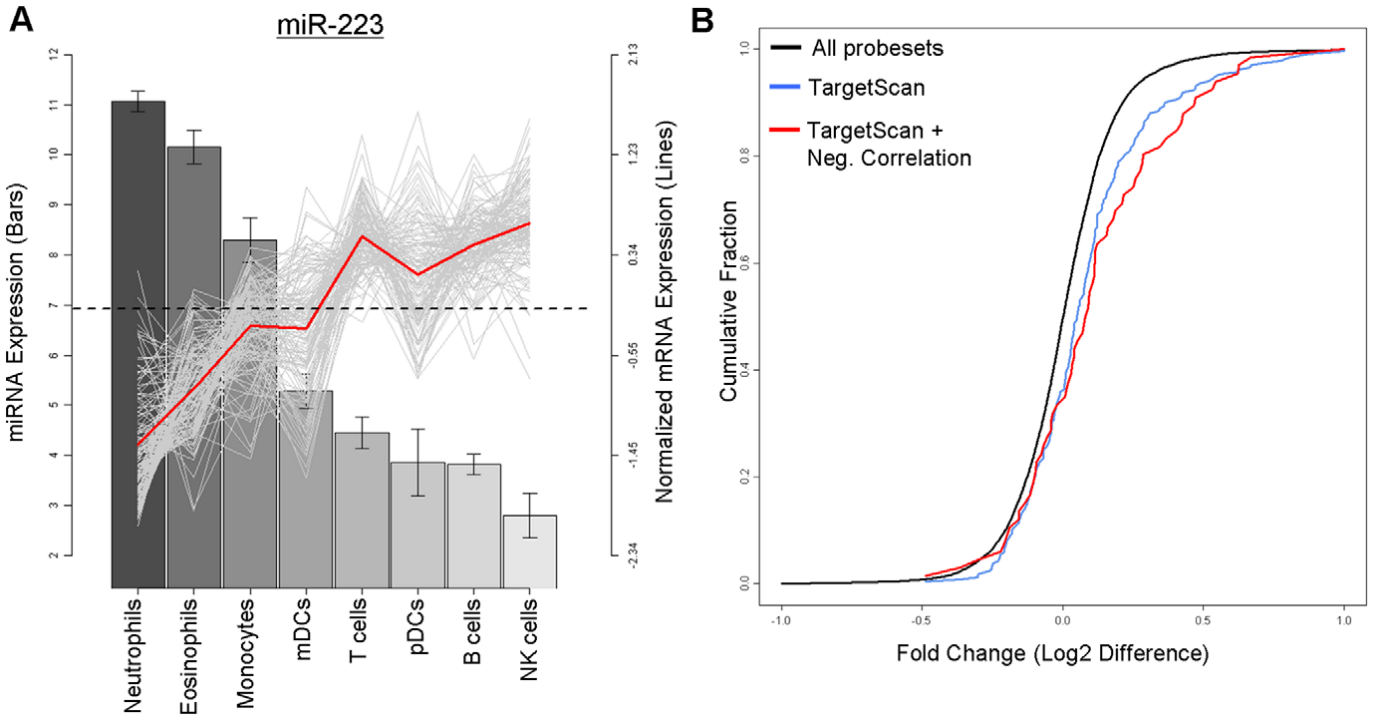
Potential miR-223 targets are repressed in a miR-223 −/− system. A) miR-223 expression across the profiled cell types (bars) is plotted against the relative expression profile (lines) of 82 genes identified as potential miR-223 targets (TargetScan, significant negative correlation). Red line represent mean expression profile of target genes, dotted line represents mean expression across cell types. B) 82 genes were identified in our study as being significant miR-223 targets. We used the data from a previously published miR-223 −/− system [25] to see if those targets would correspondingly be de-repressed when miR-223 is knocked-out. 62 of these 82 genes had matching mouse homologs (in red). The change in expression of these genes was compared against all TargetScan predicted miRNA target genes, which included predicted targets not negatively correlated with miRNA expression in our dataset (234 genes, in blue). Fold-change for all probe sets is also plotted in this figure as a null distribution (black).

To further support our observations, we searched for studies in which cell-type specific miRNA expression levels were altered by either over-expression or knock-out, and asked if the genes we had identified as miRNA targets were correspondingly up- or down-regulated in response. For miR-223, we found one study by Baek *et al.* [25], where the authors investigated the role of miR-223 in granulopoiesis by performing gene expression and proteomic profiling of mouse neutrophils obtained from a miR-223 knockout mouse vs a wild type control. Since miR-223 levels were depleted in the knockout, we asked if the 82 genes identified as miR-223 targets from our current dataset would correspondingly be up-regulated in absence of negative regulation by miR-223. 66 of these 82 genes had matching mouse homologs. By querying against the Baek dataset, we observed that 11 of 66 genes (16.7%) were indeed up-regulated, four of them (STIM1, FUBP3, CBX5, FBXO8) with fold change >1.5, the remaining seven (KPNA3, TMEM64, LRCH1, SMARCD1, PURA, RASA1, TNRC6B) with fold change >1.3 (Figure 3A).

Genes identified as miR-223 targets using TargetScan were mildly up-regulated in response to miR-223 knockout, compared to all other genes. However, genes negatively correlated with miR-223 expression in our dataset were significantly more correlated (fold change >1.3) than similar sized groups of genes randomly selected from the set of TargetScan predictions (FDR *p*<0.01, 1000 permutations) (Figure 3B), suggesting that additional filtering for negative correlation between miRNA and mRNA expression in our dataset yields an enriched set of miR-223 targets that has higher probability of being regulated by miR-223 *in vivo*.

### Validation with independent dataset

**Figure 4:**
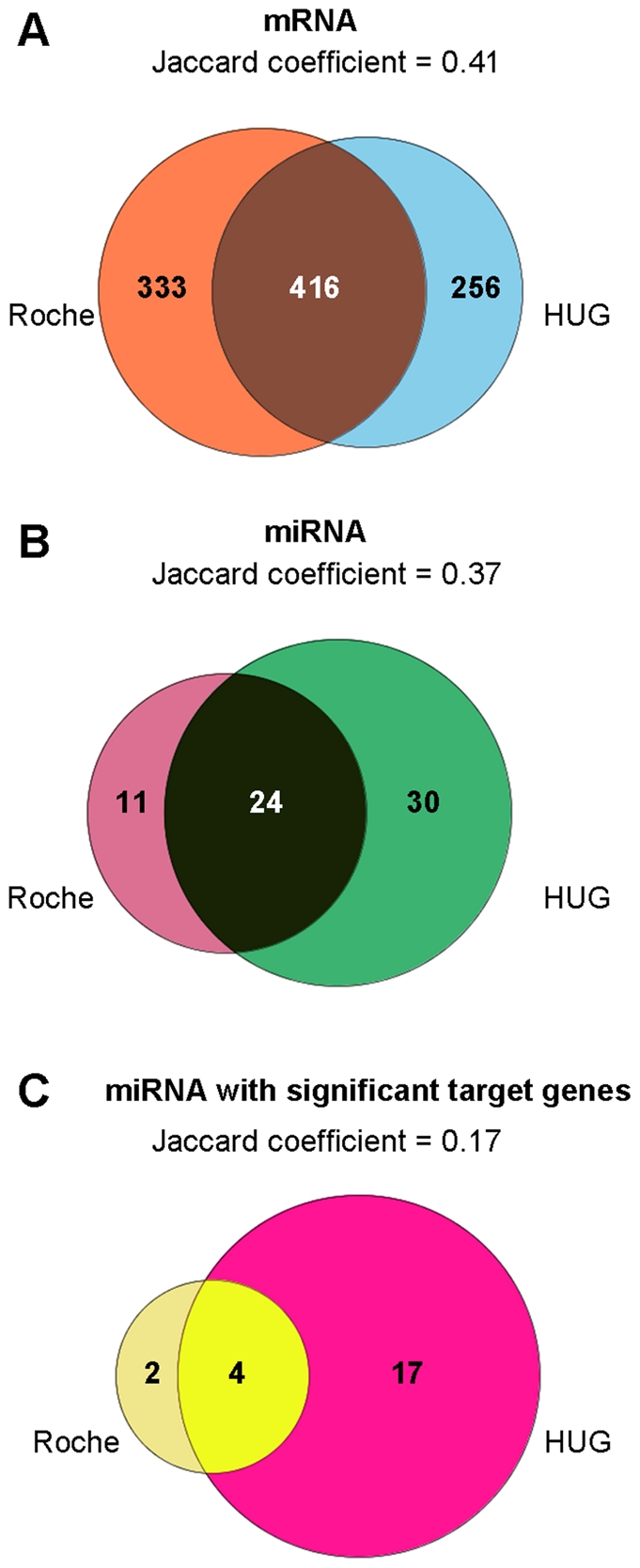
Significant overlap observed between Roche and HUG datasets. A) Excluding mDCs and pDCs, 749 genes were identified as cell-type specific in the Roche dataset, compared to 672 in HUG dataset. 416 genes were common to both (*p*<2.2e-16). The Jaccard coefficient (i.e. the intersection to union ratio), which measures sample set similarity, is 0.41. B) Excluding mDCs and pDCs, 35 miRNAs were identified as cell-type specific in the Roche dataset, compared to 54 in HUG dataset. 24 miRNAs were common to both (*p*<2.2e-16) with a Jaccard coefficient of 0.37. C) 6 miRNAs were significantly negatively correlated with their TargetScan predicted target genes in the Roche dataset, compared to 21 in the HUG dataset. 4 miRNAs were common to both datasets with a Jaccard coefficient of 0.17.

As additional validation, an independent set of seven cell subsets (CD16+CD66b+ Neutrophils, CD16−CD66b+ Eosinophils, CD14+ Monocytes, CD4+ T cells, CD8+ T cells, CD56+ NK cells and CD19+ B cells) were obtained from a separate panel of healthy donors at the University Hospital of Geneva (HUG) (Table S2), using the same protocols for cell isolation and sample processing as the Roche dataset. Samples were profiled for miRNA and mRNA expression using the same protocols and were subject to an identical data analysis workflow. This approach yielded a total of 54 miRNA probesets specifically up-regulated in one, two or three cell types in the HUG dataset, with 12 miRNA probesets previously identified as cell-type specific in the Roche dataset (The Jaccard coefficient, i.e. the intersection to union ratio, which measures sample set similarity was 0.17.) If mDCs and pDCs were excluded from the Roche dataset, the number of cell-type specific miRNA probesets common to both HUG and Roche datasets increased to 24 with Jaccard coefficient of 0.37. (Figure 4, Table S5). All miRNAs reported as specific to single cell types in the Roche dataset (miR-143, miR-31, miR-935 and miR-378) remained significant in the HUG dataset, while additional miRNAs were found to be cell-type specific, most notably miR-145 in neutrophils, miR-181 in NK cells and miR-146 in T cells and NK cells. Analysing the HUG mRNA data set in a similar way as shown above for the Roche data, we identified 672 genes specifically expressed in one, two or three cell types, of which 416 genes were also reported as cell-type specific in the Roche dataset (with both mDC and pDC samples removed to enable direct comparisons). Also similar to the Roche dataset, a large majority of genes specifically down-regulated in one cell type were repressed in neutrophils (193 genes, or 91% out of a total of 212 genes).

Next, we applied the same analysis workflow to identify genes both predicted to be miRNA targets by TargetScan and significantly negatively correlated with miRNA expression in the HUG dataset (correlation<−0.55, FDR 1%). The genes identified corresponded to predicted targets of 110 miRNAs, of which 21 miRNAs were specifically expressed in one, two or three cell types in the HUG dataset (Table S6). Of interest, miR-223, miR-143, miR-150 and miR-652 replicated as significant in both Roche and HUG datasets, strengthening the case that these miRNAs may indeed regulate expression of their respective predicted target mRNAs.

### Construction of a miRNA-mRNA regulatory network

**Figure 5:**
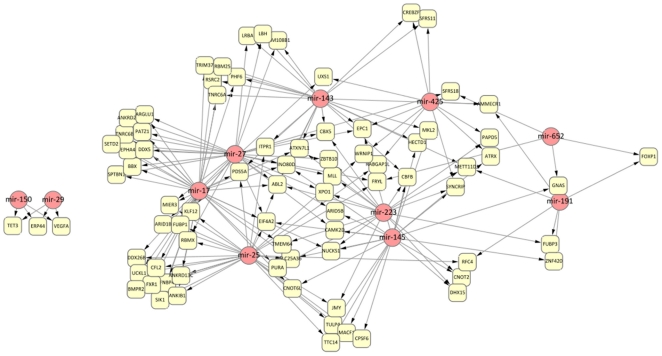
Regulatory network identifying genes that are shared targets of cell-type specific miRNAs. Networks depicting regulatory interactions between miRNAs and their respective target genes. miRNAs are coloured red, whereas target genes are coloured yellow. An edge from a miRNA node to a mRNA node indicates that the gene is both predicted to be a miRNA target by TargetScan and significantly negatively correlated with miRNA expression across the profiled blood cell subsets.

Since we observed that several miRNAs were expressed in the same cell type, e.g. miR-143, miR-145 and miR-223 are specifically expressed in neutrophils, we asked if miRNAs specific to the same cell type would target an overlapping set of genes. A gene could thus be targeted by multiple miRNAs as part of a coordinated regulatory mechanism to ensure repression of the eventual gene product. Furthermore, if these miRNAs were cell-type specific, targeting by multiple miRNAs could also imply the presence of redundant mechanisms to repress certain genes to ensure lineage commitment. To identify the genes targeted by multiple miRNAs, we compared miRNAs with significant numbers of negatively correlated target mRNAs from both Roche and HUG datasets. To obtain a higher confidence set, we selected only miRNA/target mRNA pairs that replicated across both Roche and HUG datasets for analysis, and further filtered this list for cell-type specific miRNA expression in the HUG dataset. (The HUG dataset was chosen, since it contained a greater number of cell type specific miRNAs compared to the Roche dataset). We filtered this list for genes targeted by more than one miRNA. In each case where a gene was targeted by multiple miRNAs, genomic coordinates of each seed site match were checked for overlap, to ensure that the miRNAs were not targeting the same gene due to seed site similarity (the full list of genes with respective seed site coordinates is in Table S7). The resulting miRNA/target mRNA pairs were organized in a network using Cytoscape [26], [27] where edges between miRNA and mRNA nodes represent a potential regulatory relationship. The layout of the network was determined using a spring embedded algorithm, which placed the most highly connected (multiply targeted) mRNAs in the center of the network while relegating mRNAs with fewer connections to miRNA nodes to the periphery (Figure 5). The final network consisted of two clusters, a large cluster consisting of 9 miRNAs specific to neutrophils, monocytes and myeloid lineage cells (miR-223, miR-143, miR-145, miR-25, miR-27, miR-425, miR-17, miR-652 and miR-191) and a much smaller cluster specific to lymphoid lineage cells (B, T and NK cells), consisting of miR-150 and miR-29 co-regulating TET3, ERP44 and VEGFA.

Ranking genes in the myeloid cluster by their connectivity, we found ABL2, a gene which regulates cytoskeleton remodeling during cell differentiation, cell division and cell adhesion was found to be targeted by the largest number of miRNAs (N = 5: miR-223, miR-143, miR-25, miR-27, miR-17), followed by EIF4A2, EPC1 and INO80D, which were targeted by four miRNAs each. EIF4A2 is involved in regulation of translation initiation, whereas EPC1 and INO80D are chromatin remodeling factors, involved in transcriptional regulation. To determine if other genes targeted by multiple miRNAs have similar regulatory functions, we performed Gene Ontology functional enrichment analysis on 76 mRNAs targeted by at least 2 myeloid specific miRNAs. Indeed, a large fraction of these miRNAs (32 of 76) were associated with regulation of expression (GO:0010468, *p*<9.0E-7, corrected for multiple testing), suggesting that while the myeloid specific miRNAs may be few in number (9 miRNAs), they may exert significant secondary effects on the transcription of other genes by directly repressing expression of genes involved in transcriptional regulation.

## Discussion

In this study, we sought to investigate the regulatory role of miRNAs in nine immune cell subsets found in peripheral blood. We performed miRNA profiling on a panel of nine human immune cell subsets (neutrophils, eosinophils, monocytes, B cells, NK cells, CD4 T cells, CD8 T cells, mDCs and pDCs) to identify cell-type specific miRNAs and identified four, five and nine miRNAs specific to one, two and three cell types, respectively. We then profiled mRNA expression on the same cell subsets from the same donors to investigate whether the cell-type specific miRNAs down-regulated their predicted target genes and found six cell-type specific miRNA that negatively correlated with expression of their predicted target genes. These results were validated using an independent cohort where miRNA and mRNA profiling was conducted on similar immune cell subsets (HUG dataset). To further elucidate the functional role of these miRNAs, we constructed cell-type specific regulatory networks from these miRNA/mRNA regulatory pairs. Two principal regulatory networks were found: one network consisting of 9 miRNAs from myeloid lineage cells and a second network consisting of 2 miRNAs from lymphoid lineage.

Although several groups have measured miRNA expression in selected immune subsets in previous studies, the primary focus of the present investigation was to systematically characterize cell-type specific miRNA expression and its potential impact on transcription of protein-coding genes. There were relatively few miRNAs that were found exclusively in a single cell type. We identified miR-143 as neutrophil specific, miR-31 as T-cell specific, miR-378 as monocyte specific and miR-935 as eosinophil specific. Several previous reports have shown that miR-143 is indeed expressed in neutrophils, and patients with polycythemia vera have elevated miR-143 expression in their neutrophils [28]. miR-143 and miR-145 are located in close proximity at chromosome 5q32 and while the pre-miRNA structure has not been identified, it is suggested that they are co-transcribed [29]. Indeed, we observed that miR-145 was also mainly expressed in neutrophils (fold change = 4.53 in neutrophils vs eosinophils) even though the difference was not significant. Specificity of miR-31 to T cells appears to be consistent with its role in T cell lineage determination. As reported by a previous study, miR-31 inhibits differentiation of naïve T cells to T regulatory cells by negatively regulating FOXP3 expression, binding directly to its potential target site in the 3′ UTR of FOXP3 mRNA. Consequently, miR-31 is down-regulated in nTreg compared to naïve CD4^+^CD25^−^ T cells [30], permitting FOXP3 expression and establishment of T regulatory cell identity. Little is known regarding the function of miR-378 in monocytes, or miR-935 in eosinophils.

Interestingly, miRNAs specific to three cell types displayed an expression pattern corresponding to the lineage of the cell types: lymphoid (T cells, B cells, NK cells and pDC) and myeloid (monocytes, neutrophils, eosinophils and mDC). miR-223 was an example of a miRNA expressed in myeloid cells, which agreed with previous studies showing that miR-223 was involved in the differentiation of myeloid precursors into granulocytes. miR-223 mutant mice also have higher numbers of granulocyte progenitors in the bone marrow and hypermature neutrophils in the circulation [31]. On the other hand, miR-150 was found to be specific in lymphoid cells, also confirming previous reports that miR150 is highly expressed in mature B and T cells and can be detected at high levels in the lymph nodes, spleen and the thymus [32]. The divergence in expression profiles observed between the cell subsets of distinct lineage origin reinforces the hypothesis that miRNAs are key regulators of hematopoiesis.

By performing mRNA expression profiling of immune cell subsets isolated from the same donors, we identified genes that also displayed similar specificities to one, two or three cell types and attempted to correlate the expression of these genes to miRNA expression profiles. We found 542 genes specific to a single cell type, with 456 genes up- and 86 genes down-regulated. Unlike the up-regulated genes, genes specifically down-regulated in a single cell type were predominantly repressed in neutrophils. Neutrophils are short-lived cells playing an important role in inflammatory reactions, with a half-life of 6 to 7 hours in blood. This short life span might explain why neutrophils have poorly developed machinery for protein synthesis, and why genes related to transcription and protein synthesis (i.e. ribosomal protein genes) are profoundly under-represented. Despite this, neutrophils maintain active genes responsible for their unique activities and are able to synthesize RNA and proteins *de novo* following activation [33]. A recent study by Rossi et al. describes the miRNA-ome of most human lymphocyte subsets [34]. miR-125a-5p, which we found in our study specifically expressed in T cells, was also found to be expressed by CD4 and CD8 cells, in the study of Rossi et al. In concordance with their results we also found expression of miR-150 in all B, T and NK cells subsets. Finally, they found miR-125b to be highly specific for naïve CD4 T cells and to constitute an important regulator of the naïve state of those cells; we found this miRNA to be specifically expressed in CD4 and CD8 cells in the lymphocyte compartment.

In correlating miRNA expression with expression of their predicted mRNA targets, we identified 94 miRNAs that were likely negative regulators of their respective mRNA targets. Of these, six miRNAs were cell-type specific, suggesting that these miRNAs could play a functional role in defining the cell-type specificity of their respective targets. One of these miRNAs was miR-223, for which we were able to show that the target genes identified were correspondingly up-regulated in neutrophils of a miR-223 gene knockout mouse [25], serving as additional support that these genes may indeed be modulated by miR-223 *in vivo*. This study also showed that mRNAs of most of the highly responsive proteins were de-repressed already at the myeloid progenitor stage, suggesting that miR-223 is important already early in neutrophil differentiation.

As further validation of our approach, we decided to profile the same cell subsets in an independent test cohort recruited at the University Hospital of Geneva (HUG). When the same analysis workflow was applied to the HUG dataset, we identified 54 cell type specific miRNAs, of which 21 were significantly negatively correlated with expression of their predicted target genes. Analysis of the HUG dataset yielded more significant miRNAs and mRNAs than the Roche dataset, but this was likely due to two reasons: firstly, the HUG dataset did not include mDCs or pDCs, which reduced the total number of cell types a miRNA or mRNA transcript had to be compared against in order to be identified as cell-type specific. When mDCs and pDCs were removed from the Roche dataset to ensure direct comparison across datasets, the agreement between both datasets increased, as measured by the Jaccard coefficient, from 0.29 to 0.41 for the mRNA data and from 0.17 to 0.37 for the miRNA data. Secondly and perhaps more importantly, samples from each cell type in the HUG dataset were hybridized to microarrays and run on separate days. We assumed that the biological difference between cell types would be greater than the technical variation in array quality across scan dates; however this lack of randomization could have artificially increased the apparent difference in miRNA expression between cell types. Scan date and cell-type variability in the HUG miRNA and mRNA datasets were visualized using principal components analysis (PCA) (Figure S5). Since each cell type was scanned on a separate date, the scan date effect was completely confounded with the cell type effect and could have contributed to additional separation between samples in the PCA plot. Overlap in terms of the number of differentially expressed miRNAs could be optimized via a maximum likelihood approach as developed by Git et al [35]. In their iMLE algorithm, the authors varied significance thresholds for each dataset iteratively to obtain the highest number of differentially expressed ‘truth’ calls across all compared datasets or platforms. We attempted to apply this method to the current comparison between Roche and HUG datasets, but were unable to obtain relevant results, most likely because only 2 datasets were being compared as opposed to 6 independent datasets from different platforms in Git et al.

miRNAs can act via two mechanisms to cause target gene repression: translational inhibition and transcript destabilization and degradation. Transcript destabilization and degradation is favored when complementarity between the miRNA and the seed site match in the 3′ UTR increases. Indeed, by using negative correlation between miRNA and mRNA expression to identify miRNA target genes, we tacitly assume that these miRNA predominantly act on their targets by favoring the second mechanism: transcript destabilization and degradation. It remains to be determined what fraction of miRNA targets are solely regulated by this mechanism, although a recent study using ribosome profiling suggested that miRNAs predominantly act in mammalian cells through decreasing levels of target mRNAs [20]. Another possibility might be that the inverse expression patterns we observed between miRNAs and their predicted mRNA targets are due to indirect regulation, which is impossible for us to rule out but appears highly unlikely.

While the assumption that miRNAs regulate target mRNA expression via transcript degradation may not be true in general, in cases where a gene is targeted by multiple miRNAs, cooperative action between multiple miRNAs could favor transcript degradation as opposed to translational inhibition. Towards this end, we constructed a regulatory network of cell-type specific miRNAs and their putative target genes, which enabled us to identify target gene “hubs”, or genes targeted by multiple miRNAs, which increased their likelihood of being targeted for transcript degradation. The regulatory network consisted of a small cluster of miRNAs specific to lymphoid lineage cells (miR-150 and miR-29) and a much larger cluster of miRNAs specific to myeloid cells. We found ABL2 to be targeted by the largest number of myeloid specific miRNAs. This was consistent with reports of ABL2 fusion transcripts in AML cell lines [36], which suggested that increased activity of transformed ABL2 helped maintain an undifferentiated myeloid precursor state. Myeloid specific miRNAs may promote differentiation and granulopoiesis by repressing ABL2 levels, disrupting maintenance of the undifferentiated myeloid precursor state. Targeting of ABL2 by various miRNAs could be a redundant mechanism to ensure ABL2 repression in myeloid cells, and it would be interesting to study whether indeed ABL2 levels could be modulated in a dose dependent fashion *in vivo* by perturbing the expression of one or more of its regulatory miRNAs.

The organization of myeloid miRNAs in the regulatory network placed miR-223, miR-143 and miR-145 in a central position, since they targeted several genes in common with other miRNAs. This suggests that they might play a determinant role in cell-fate decision during granulopoiesis. Indeed, miR-223, miR-143 and miR-145 have been shown to be overexpressed in myeloid cells from polycythemia vera patients in which enhanced erythropoiesis is observed [28], suggesting that these 3 miRNAs are usually decreased during erythropoiesis. It would be very interesting to study their expression in common myeloid progenitors and how their regulation plays a role in erythropoiesis versus granulopoeisis differentiation.

In conclusion, our study identifies miRNAs, their respective target genes, and the regulatory role of miRNAs in specific immune cell subsets. To our knowledge, it is the first study attempting to simultaneously profile mRNA and miRNA from a diverse panel of immune cells in a systematic manner. We have identified strong candidates for miRNA/mRNA regulatory relationships specific to myeloid and lymphoid cell subsets, which contribute to our understanding of how miRNA-mediated regulation can impact the establishment of immune cell identity.

## Methods

### Ethics Statement

This study was performed in conformity with requirements approved by the institutional review boards of F.Hoffmann-LaRoche Ltd and the University Hospital of Geneva. This study was approved by the institutional review boards at F.Hoffmann-LaRoche and the University Hospital of Geneva. All participants were recruited following written informed consent.

### Cell isolation

Peripheral blood was obtained from healthy donors with ages ranging from 15 to 59 and with equal gender distribution. Blood was drawn in EDTA tubes (BD Vacutainer, Franklin Lakes, NJ, USA). A nucleated cell suspension was prepared from 50 mls of whole blood using lymphocyte separation medium (LSM, MP Biomedicals, LLC Solon, OH, USA). The cell pellet was transferred to a new tube and lysed with ammonium chloride solution (PharMLyse, BD Biosciences, Breda, The Netherlands) on ice for 10 minutes. After washing the pellet twice with PBS-2%FBS-1 mM EDTA, cells were resuspended at 5×10^7^/mL and neutrophils and eosinophils were isolated using the EasySep® Human Neutrophil Enrichment Kit (Stem Cell Technologies, Grenoble, France) and EasySep® Human Eosinophil Enrichment Kit (Stem Cell Technologies) respectively according to manufacturer's instruction. The PBMCs fraction was washed twice with PBS-2%FBS-1 mM EDTA. CD14+ monocytes, CD8+ T cells, CD56+ NK cells and CD19 B cells were isolated using the Human CD14 positive Selection Kit, Human CD8+ T cells Enrichment Kit, EasySep® Human CD56+ NK cells Enrichment Kit, EasySep® Human CD19+ B cells Enrichment Kit (all from Stem Cell Technologies), respectively, according to manufacturer's instructions. The CD4+ T cells were isolated from the monocyte-depleted fraction (obtained during the monocyte isolation) using the EasySep® Human CD4 Positive Selection Kit (Stemcell Technologies) according to manufacturer's instructions. Blood dendritic cells were obtained from a second batch of experiments. Briefly, a nucleated cell suspension was prepared from EDTA treated whole blood (∼50 mls) using lymphocyte separation medium (LSM, MP Biomedicals, LLC). The PBMC fraction was washed twice with PBS-2%FBS-1 mM EDTA and CD123+ plasmacytoid dendritic cells (pDCs), CD11c+ myeloid dendritic cells (mDCs) and CD14+ monocytes were isolated using the EasySep® Human pDC Enrichment Kit, a custom EasySep® Human mDC Enrichment Kit (containing antibodies directed against CD3, CD19, CD14, CD16, CD19, CD25, CD34, CD56, CD66b, GlyA, CD85g, CD45RA, CD123 and dextran) and the Human CD14 positive Selection Kit (all from Stemcell Biotechnologies), respectively, according to manufacturer's instructions. When indicated, PBMCs or granulocytes from 5 donors were pooled followed immediately by cell subset isolation. The incubation time of cells from different donors origin was kept to a minimum (never exceeding 30 minutes at 4°C) in order to minimize cell activation. The purity of the isolated cell population was assessed by FACS using the following antibodies: anti-CD3 AF488, anti-CD66b FITC, Lin FITC, anti-CD14 PE, anti-CD56 PE, anti-CD123 PE, anti-CD45 PE-Cy7, anti-CD8 PerCP, anti-HLA-DR PerCP, anti-CD19 APC, anti-CD11c APC, anti-CD4 APC-Cy7, anti-CD16 APC-Cy7 (all from BD Bioscience), and was >90% for CD4+ T cells and mDCs and >95% for all other cell types.

### RNA isolation

Total RNA, including small non-coding RNAs, was extracted using the mirVana RNA isolation kit (Ambion, Inc, Austin, TX, USA), according to manufacturer's protocol. RNA was quantified using a ND-1000 spectrophotometer (Nanodrop Technology®, Cambridge, UK) and RNA integrity was assessed using the Agilent 2100 Bioanalyzer (Agilent technologies, Santa Clara, CA, USA). The 260/280 ratio were between 1.7 and 2.1 and the RINs (RNA Integrity Numbers) were >6 for all samples.

### Microarray processing

For mRNA expression analysis total RNA (100 ng) was transcribed and labeled into amplified RNA (aRNA) using the 3′IVT express kit (Affymetrix Inc., Santa Clara, CA) according to manufacturer's instruction on a GCAS (Gene Chip Array Station). Fragmented and labeled aRNA was hybridized to the Affymetrix U133 Plus 2.0 chips, according to manufacturer's instructions.

For miRNA expression analysis total RNA (300 ng) was biotin labeled using the FlashTag™ HSR RNA labeling kit for Affymetrix miRNA arrays (Genisphere LLC., Hatfield, PA, USA), according to manufacturer's instructions. Labeled RNA was hybridized to Affymetrix GeneChip miRNA microarrays, according to manufacturer's instructions. The data is available in GEO under the accession number GSE28492.

### Data analysis

The quality of the raw data (CEL files) was assessed in Bioconductor using 4 metrics: maplot, spatial, boxplot and rle. Data was then normalized using the robust multi-array average (RMA) expression measure [37]. Probesets were then annotated using the latest annotation available in Bioconductor and curated using an internal Roche probe inspector tool, which updates gene annotation per probeset based on the latest human genome draft release. As an additional quality measure, probesets were also curated using the tool for sequence specificity; probesets mapping to non-unique locations in the genome were flagged and ignored in subsequent analysis. A total of 20,367 out of 54,675 probesets passed the QC and curation process, corresponding to 11,629 unique genes. Using principal component analysis (PCA) to visualize sample-to-sample variability in miRNA data, we flagged 3 mDC samples (donor pools 16, 17 and 18) as outliers and excluded them from subsequent analysis. 4 samples from the June 22^nd^ 2010 scan date were also excluded as outliers (CD4+ T cell sample from donor pool 2, CD14+ monocyte sample from donor pool 7, eosinophil sample from donor pool 3 and neutrophil sample from donor pool 7). For the mRNA data, 1 eosinophil (donor pool 1) and 2 neutrophil samples (donor pools 1 and 2) were also flagged as outliers and removed from analysis.

### Identification of cell-type specific miRNA and mRNA transcripts

For miRNA analysis, probesets were filtered for detection by requiring RMA signal value to be at least greater than 7 in at least one cell type (considered above detection limit). A total of 8 immune cell subsets were considered in the analysis: T cells (CD4+, CD8+), B cells (CD19+), NK cells (CD56+), Monocytes (CD14+), Neutrophils, Eosinophils, mDCs and pDCs. CD4+ and CD8+ subsets together as ‘T cells’ because few transcripts were unique to resting CD4+ cells or CD8+ cells only. Transcripts with significant differential expression across cell subsets were identified using ANOVA. For each transcript, we ranked the various cell-types in descending order, i.e. from highest-expressing to lowest-expressing. miRNAs specifically up or down regulated in 1,2 or 3 cell-types were identified by requiring significant difference in expression between the highest expressing cell types (or lowest for cell type specific down-regulation) vs. the next highest cell type (*p*<0.05, with Benjamini-Hochberg correction for multiple testing). A fold-change cutoff was not imposed as a criterion for cell type specific expression since few miRNAs displayed marked fold-change differentials from highest/lowest expressing group relative to next highest/lowest expressing cell type.

For mRNA analysis, transcripts were identified as specifically up-regulated in 1, 2 or 3 cell types if differences in expression within the top group were insignificant, whereas difference relative to the next highest expressing cell-type was at least 4 fold. (*p*<0.05, with Benjamini-Hochberg correction for multiple testing). Transcripts specifically down-regulated in 1, 2 or 3 cell types were identified by applying a similar set of criteria to the lowest expressing 1, 2 or 3 cell-types.

In both miRNA and mRNA analysis, CD4+ and CD8+ T cells were considered as a single group: ‘T cells’ because few transcripts were unique to CD4+ cells or CD8+ cells only. For example, the only mRNA transcript specific to CD8 T cells was CD8B. CD8A was expressed by CD8+ T cells and NK cells with a higher level in CD8+ T cells but the difference was not significant. Three other genes previously identified to be CD8+ T cell-specific (DKK3, *CD248* and T-cell receptor alpha V gene segment TRAV1-2) [8] could not be detected in our study (signal intensity below log_2_ 7 or non-specific probeset mapping to genome).

### Platform comparison between Roche and HUG

Human Reference RNA (Agilent technologies) and First Choice Human RNA (Ambion) were used as a RNA source respectively for mRNA and miRNA platform validation between the two sites. Arrays from 6 technical replicates for mRNA and 4 for miRNA were processed at Roche and at HUG and evaluated to assess the level of comparability between the two platforms. Data were preprocessed separately for Roche and HUG arrays as described above (RMA normalization, internal probe curation and QC). In order to identify problematic arrays, QC was performed before and after pre-processing. QC checks included: Evaluation of the intensity profiles, M and A plots, between-arrays comparisons of average background, scale factor, GAPDH 3′/5′ ratios, RNA degradation (based on RNA digestion plots), PLM (probe level models) methods (e.g. RLE, NUSE) and multivariate expression profiling obtained by principal component analysis (PCA) available in the Bioconductor *affy* package in the R programming environment.

### Correlation of miRNA and target mRNA expression profiles

Expression profiles for all 20,367 probesets and 1,801 human miRNA probes were Z-score transformed to have zero mean and unit variance across cell-types. To identify miRNAs that significantly negatively correlate with target mRNA expression, we computed the Pearson correlation for each miRNA/mRNA probeset pair. Pairs displaying significant negative correlation (<−0.55) were selected for further analysis. The value for the correlation cutoff (−0.55) was determined by setting a FDR of 1% cutoff on the results of 100 permutations - in each permutation, cell-type labels were randomized and miRNA-mRNA pairwise correlations recomputed.

Target genes for miRNA were predicted using a Roche modified TargetScanS algorithm and further cross-checked with predictions from 2 other algorithms (miRanda and miRDB) and experimentally validated targets from mir2disease and from tarbase. Overlap between mRNAs significantly negatively correlated with miRNA expression and mRNAs predicted to be miRNA targets were calculated for each miRNA. Significance of overlap was quantified by performing 100 permutations to estimate false discovery rates. The number of predicted mRNA targets per miRNA was fixed in each permutation, while their identities were randomized. FDR cutoffs of 1% and 5% were applied to obtain lists of miRNAs with significant numbers of predicted mRNA targets that are also negatively correlated in expression.

## Supporting Information

Figure S1 **Clustering of single donor and pooled donor samples based on correlation of miRNA expression profiles.** Samples from single or pooled donors are listed in the same order on x and y axes. Samples from pooled donors are indicated by “Pool” followed by the donor pool number, cd19: B cells, cd56: NK cells, cd4: CD4+ Tcells, cd8: CD8+ T cells, cd14: Monocytes, eosino: Eosinophils, neutro: Neutrophils. Samples from single donors are indicated by cell type, followed by single donor index. Correlation between samples based on miRNA expression is represented by the heatmap coloring scheme, ranging from anti-correlated (Blue: −1) to correlated (Red: 1).
(TIFF)

Figure S2 **miR-31, miR-143, miR-223 and miR-150 are confirmed to be cell type specific, using data from single donor samples.** Expression levels for miR-31, miR-143, miR-223 and miR-150 (Log2, mean ± SEM) are plotted across a panel of immune cell subsets, for samples obtained from single donors (dark shaded bars) and pooled donors (light shaded bars).
(TIFF)

Figure S3 **Average correlation between single and pooled donor samples is >0.62 across cell types.** Correlations were computed between mRNA expression profiles of single and pooled donor samples, for each cell type. The distributions of correlation values for each cell type are plotted as histograms, with the average correlation listed for each cell type.
(TIFF)

Figure S4 **Clustering of single donor and pooled donor samples based on correlation of mRNA expression profiles.** Samples from single or pooled donors are listed in the same order on x and y axes. Samples from pooled donors are indicated by “Pool” followed by the donor pool number, cd19: B cells, cd56: NK cells, cd4: CD4+ Tcells, cd8: CD8+ T cells, cd14: Monocytes, eosino: Eosinophils, neutro: Neutrophils. Samples from single donors are indicated by cell type, followed by single donor index. Correlation between samples based on mRNA expression is represented by the heatmap coloring scheme, ranging from anti-correlated (Blue: −1) to correlated (Red: 1).
(TIFF)

Figure S5 **PCA plots of miRNA and mRNA data from the HUG dataset.** Principal component analysis (PCA) was performed on mRNA and mRNA expression data from the HUG cohort. Samples were colored by scan date and represented as different shapes by cell type.
(TIFF)

Table S1 **Sample information.**
(XLS)

Table S2 **Blood Donor information.**
(XLS)

Table S3 **List of 696 mRNA transcripts specific to 1, 2 or 3 blood cell subsets.**
(XLS)

Table S4 **List of 94 miRNAs significantly negatively correlated with target mRNA expression (TargetScan).**
(XLS)

Table S5 **List of 55 miRNAs which are cell-type specific in the HUG dataset.**
(XLS)

Table S6 **List of 21 miRNAs which are cell-type specific in HUG dataset and negatively correlate with target mRNA expression (TargetScan).**
(XLS)

Table S7 **List of target genes that are regulated by multiple miRNAs.**
(XLS)
