# Supplementary figures and images for: Expression Profiling of Human Immune Cell Subsets Identifies miRNA-mRNA Regulatory Relationships Correlated with Cell Type Specific Expression
Source: PLoS One. 2012 Jan 20;7(1):e29979. doi: 10.1371/journal.pone.0029979 (PMC3262799; doi:10.1371/journal.pone.0029979)

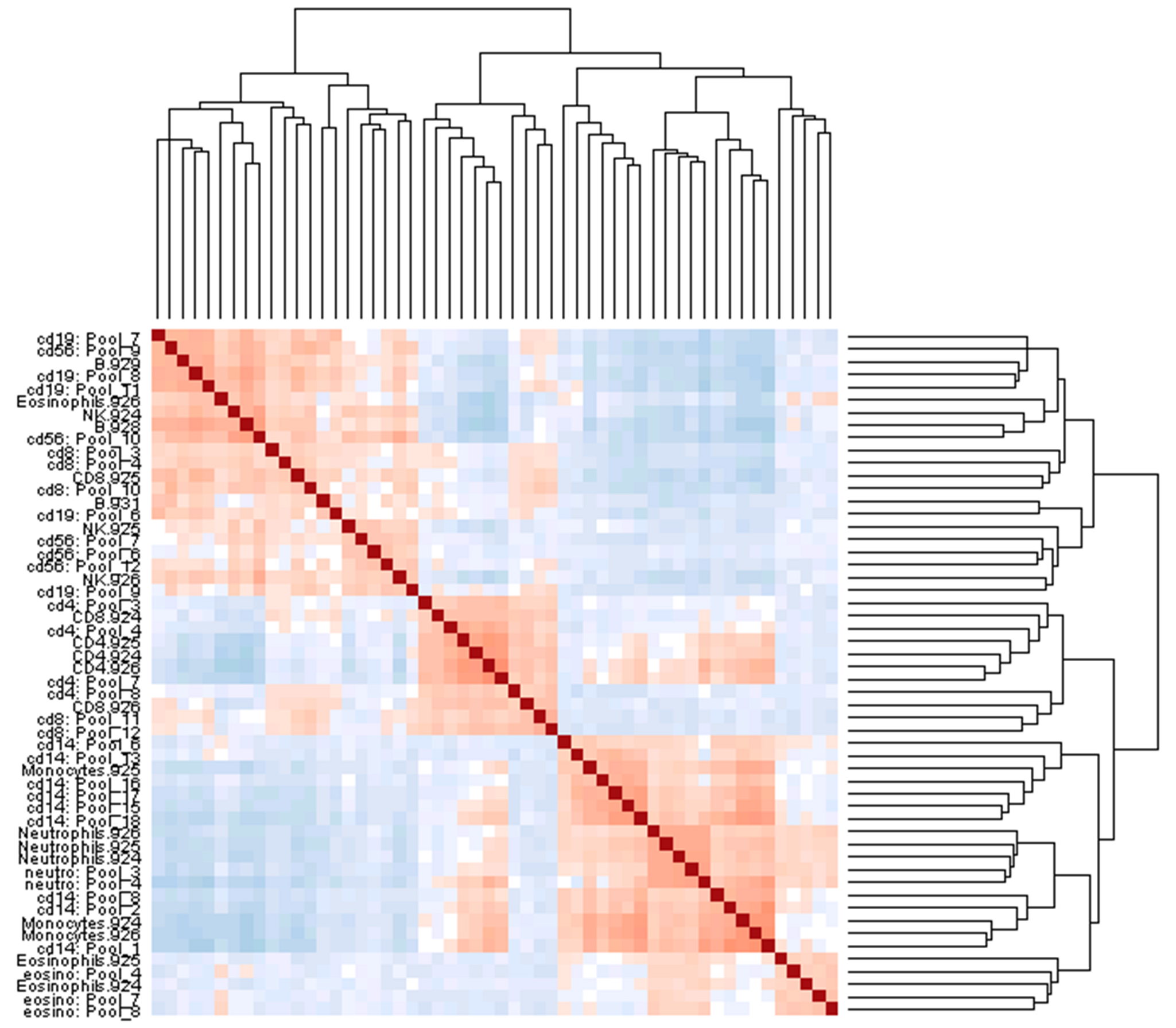

Supplement: Figure S1 — Clustering of single donor and pooled donor samples based on correlation of miRNA expression profiles. Samples from single or pooled donors are listed in the same order on x and y axes. Samples from pooled donors are indicated by “Pool” followed by the donor pool number, cd19: B cells, cd56: NK cells, cd4: CD4+ Tcells, cd8: CD8+ T cells, cd14: Monocytes, eosino: Eosinophils, neutro: Neutrophils. Samples from single donors are indicated by cell type, followed by single donor index. Correlation between samples based on miRNA expression is represented by the heatmap coloring scheme, ranging from anti-correlated (Blue: −1) to correlated (Red: 1). (TIFF) [file pone.0029979.s001.tif]

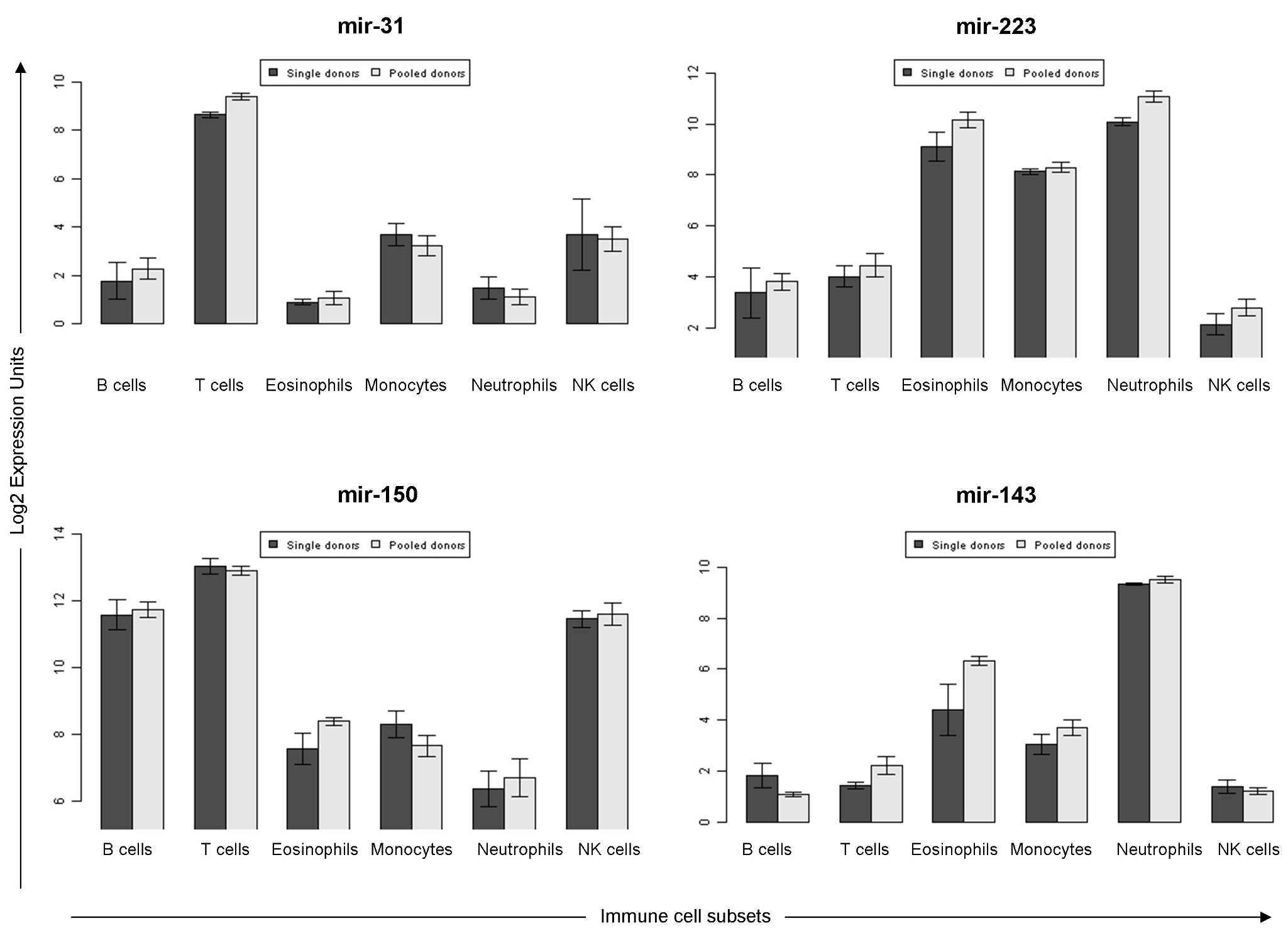

Supplement: Figure S2 — miR-31, miR-143, miR-223 and miR-150 are confirmed to be cell type specific, using data from single donor samples. Expression levels for miR-31, miR-143, miR-223 and miR-150 (Log2, mean ± SEM) are plotted across a panel of immune cell subsets, for samples obtained from single donors (dark shaded bars) and pooled donors (light shaded bars). (TIFF) [file pone.0029979.s002.tif]

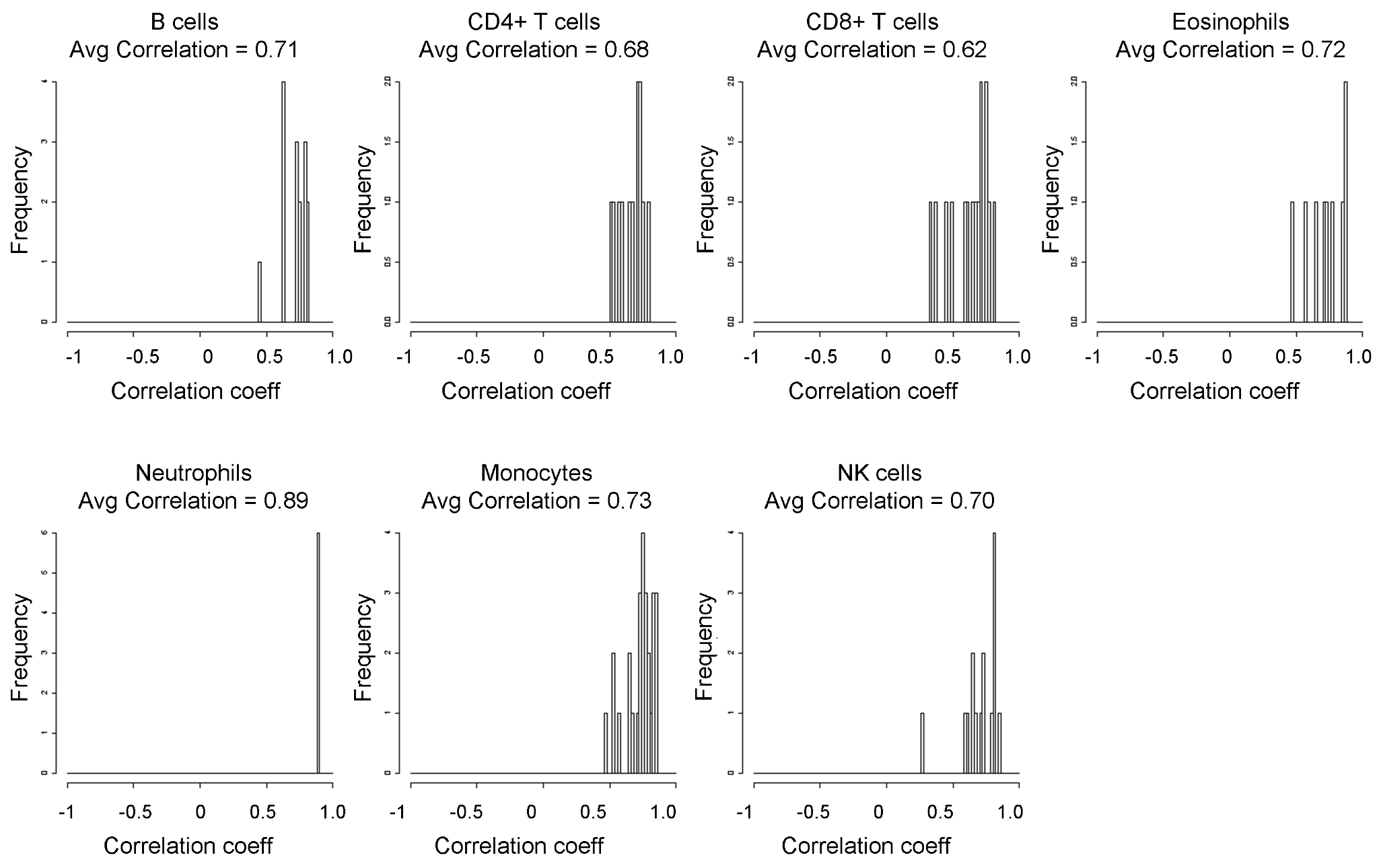

Supplement: Figure S3 — Average correlation between single and pooled donor samples is >0.62 across cell types. Correlations were computed between mRNA expression profiles of single and pooled donor samples, for each cell type. The distributions of correlation values for each cell type are plotted as histograms, with the average correlation listed for each cell type. (TIFF) [file pone.0029979.s003.tif]

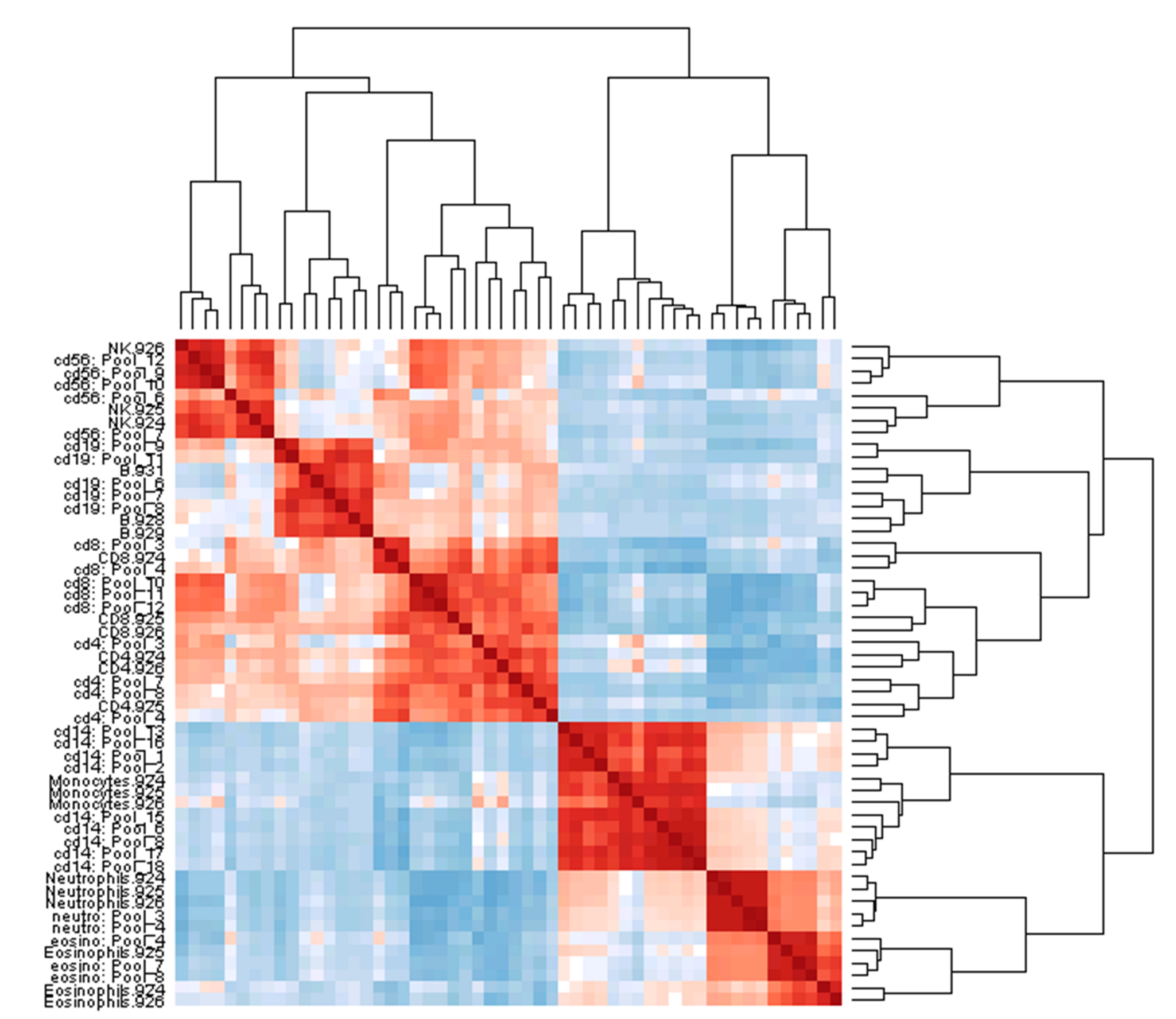

Supplement: Figure S4 — Clustering of single donor and pooled donor samples based on correlation of mRNA expression profiles. Samples from single or pooled donors are listed in the same order on x and y axes. Samples from pooled donors are indicated by “Pool” followed by the donor pool number, cd19: B cells, cd56: NK cells, cd4: CD4+ Tcells, cd8: CD8+ T cells, cd14: Monocytes, eosino: Eosinophils, neutro: Neutrophils. Samples from single donors are indicated by cell type, followed by single donor index. Correlation between samples based on mRNA expression is represented by the heatmap coloring scheme, ranging from anti-correlated (Blue: −1) to correlated (Red: 1). (TIFF) [file pone.0029979.s004.tif]

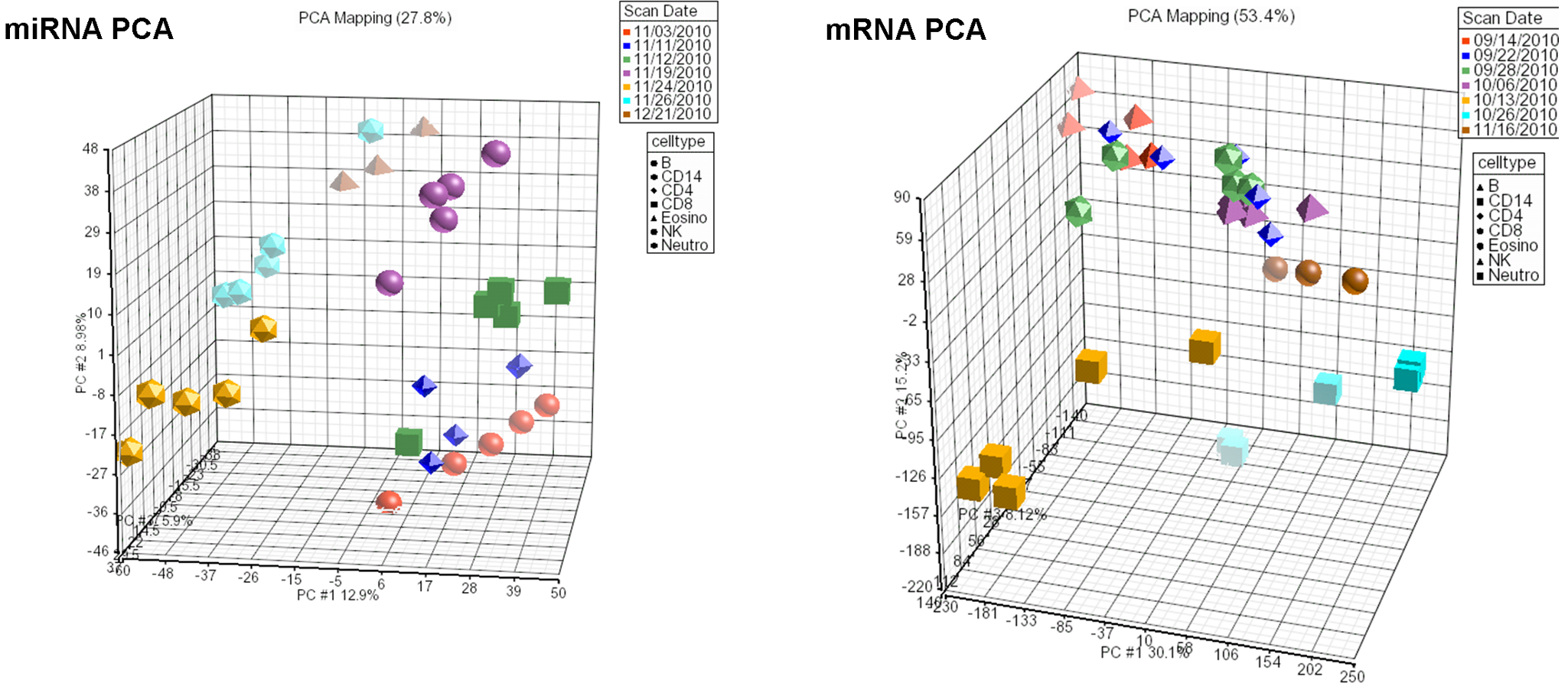

Supplement: Figure S5 — PCA plots of miRNA and mRNA data from the HUG dataset. Principal component analysis (PCA) was performed on mRNA and mRNA expression data from the HUG cohort. Samples were colored by scan date and represented as different shapes by cell type. (TIFF) [file pone.0029979.s005.tif]
